# The Contribution of Neighborhood Tree and Greenspace to Asthma Emergency Room Visits: An Application of Advanced Spatial Data in Los Angeles County

**DOI:** 10.3390/ijerph18073487

**Published:** 2021-03-27

**Authors:** Dohyung Kim, Yongjin Ahn

**Affiliations:** 1 Department of Urban and Regional Planning, California State Polytechnic University, Pomona, 3801 W Temple Ave., Pomona, CA 91768, USA; dohyungkim@cpp.edu; 2 School of Architecture, Seoul National University of Science & Technology, Nowon-gu, Seoul 01811, Korea

**Keywords:** asthma, public health, neighborhood tree, open/greenspace, spatial regression, Light Detection and Ranging (LiDAR)

## Abstract

This paper aims to investigate the role of neighborhood tree and greenspace on asthma morbidity, especially asthma emergency room visits. We employed advanced spatial data which allow for precisely capturing both the quantity and the features of tree and greenspace within a neighborhood environment. The results from the spatial regression models in Los Angeles County revealed that the features of trees and greenspace, such as the configuration of the tree canopy, the level of tree clustering, and private neighborhood greenspaces contribute to decreasing asthma morbidity, in addition to the quantity of tree and greenspace acreages. Notably, however, large scale greenspace, such as golf courses, school playgrounds, and parks fails to reduce the number of asthma emergency room visits at the statistically significant level. These findings imply that the creation of dense or clustered tree patches and small-scale neighborhood greenspaces might play a substantial role in mitigating air quality and consequently reducing asthma emergency room visits.

## 1. Introduction

Asthma is one of highly prevalent health risks with a significant influence among the US population [1]. According to the recent national statistics, as of 2018, about 7.7 percent of total U.S. population suffers from asthma. While controlled asthma has a minimal impact on everyday life, 61.9% of adult asthma patients experience uncontrolled asthma with high frequency and intensity which can lead to a significant social and health cost [1]. These patients have an increased risk of an emergency department visit, hospitalization and work and school absenteeism.

Although the causal pathways to asthma are still unconvincing, it has been generally accepted that the exposure to air pollutants outdoor, such as ozone, particulate matter (PM), and diesel exhaust, can trigger asthma attacks [2]. Given this clear connection between outdoor air quality and asthma morbidity, large volume of literature in environmental health research more related to asthma has paid attention to the important roles of greenspace in alleviating air pollutants [3,4,5,6,7].

In general, the relationship between urban green spaces and the prevalence of asthma is complicated and conditional, rather than steady and independent. For instance, some previous studies offered evidence on the health benefit of an urban green environment, arguing that greenspaces and trees can positively and protectively contribute to asthma morbidity [8,9,10]. Another cross-sectional study also verified the confounding effect of green spaces, traffic volume, and the perceived lack of area safety, in explaining the prevalence of childhood asthma [9]. However, these findings are not always consistent. A cross-sectional study revealed different roles of different types of urban green environments in asthma, depending on the level of pollutant exposure [8]. Green space and gardens contribute to decreasing asthma hospitalization, when the level of pollutant exposure is lower, whereas tree density plays a role in reducing asthma hospitalization, when the level of pollutant exposure is higher. Others did not find any supportive evidence on the hypothesized beneficial influence nor statistical significance in the association between urban green environment and asthma morbidity [11,12,13]. Normally, these previous studies present a home-based measurement of greenspace (i.e., buffer area) [12], a case-control design [13], or birth cohort data linked to detailed geographic information [11].

In summary, mixed findings or conflicting influence of urban green environments on asthma morbidity are possibly due to the diverse nature of research design. They include different measurements in urban trees/greenspaces and/or asthma outcomes, different study areas, different periods (cross-sectional- vs. longitudinal study), different target groups (observational- vs. case-control/birth-cohort study), and different units of analysis (ecological- vs. individual level). Among the variations in research design driven by the quantitative analytic approach, this study raises the measurement issue of urban trees/greenspaces.

Although considerable research has addressed the influence of greenspace on asthma, it is still questionable whether or not the greenspace data employed by the research adequately measure the area of greenspaces in the urban environment. Much research conventionally applied land cover data derived from satellites to measure greenspace. However, the resolution of the land cover data is too low to accurately capture the boundary of tree patches and greenspaces in urban environments, because of the complex and dense patterns of land use in urban areas [14]; and because of scattered small-scale greenspaces, such as backyards, front yards, and pocket parks [15]. Furthermore, the conventional satellite data are not sufficient to capture the features and configurations of greenspace and trees. Keeping in mind the fairly defined relationship between asthma and air quality, it is reasonable to presume that the features and configurations of urban tree and greenspace (e.g., the size of tree patches) are probably related to asthma morbidity. However, there has been a lack of research which adequately addresses the features and configurations, mainly due to the scantiness of suitable data with the high resolution.

Considering this research gap, this paper aims to examine how and to what extent neighborhood greenspaces and trees, measured by advanced spatial data, influence asthma morbidity, especially asthma emergency room visits. Since high-resolution spatial data allow the precise capturing of urban greenspaces and trees, this paper sheds light on the relationship of asthma morbidity, not only with the quantity of greenspaces and trees but also with their characteristics. Conducting a comprehensive measurement of the quantity, features, and configuration of neighborhood greenspace and trees in Los Angeles County, this paper constructs two spatial regression models that quantify the role of the greenspaces and trees on asthma emergency room visits, while controlling the socio- demographic and air quality factors at the census tract level.

## 2. Methodology

The focus of this study is to identify the role of a neighborhood’s tree and greenspace on asthma morbidity under the control of socio-demographic and air quality factors. A geographical area of this research is Los Angeles County, California, U.S., and we defined the census tract as the unit of analysis for an empirical test of the relationship between greenspaces/trees and asthma morbidity at the local level. There are 2346 census tracts in total within Los Angeles County, but we built a data set of analysis variables for 2301 census tracts. Since 45 census tracts only represent state parks, national forests, or mountain areas where there is no residential household, we removed those areas from the final analysis.

**Figure 1:**
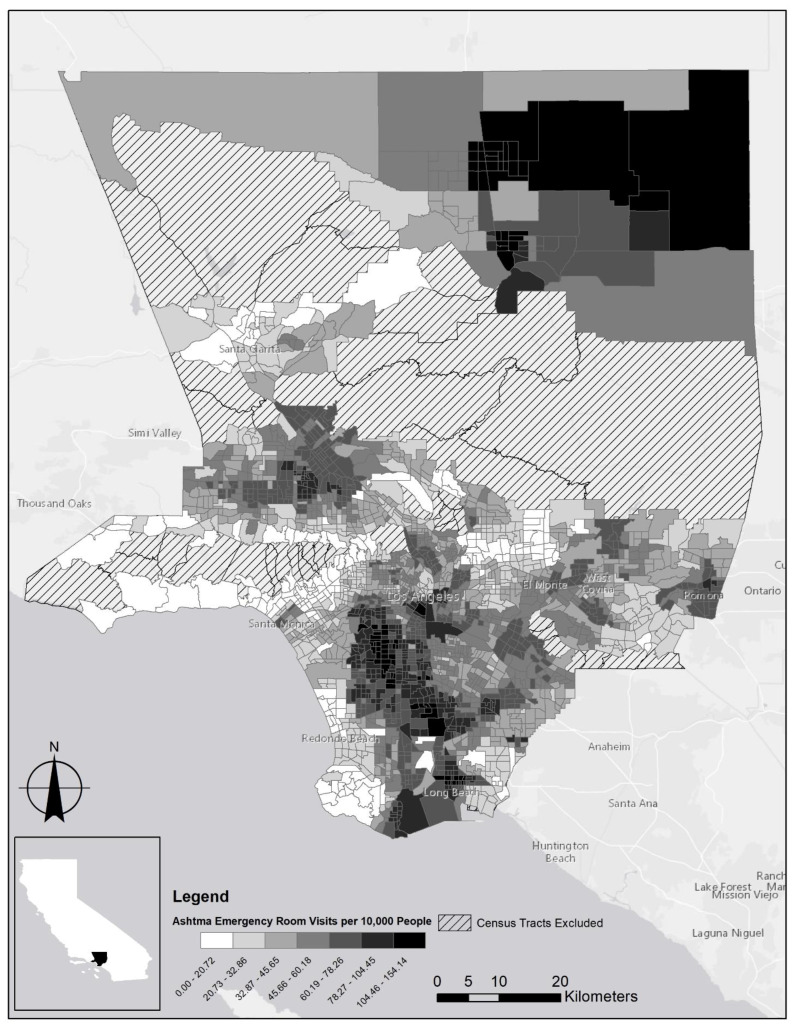
Asthma emergency room visits per 10,000 people across the Los Angeles County census tracts (2011–2013)**.**

The dependent variable of the model is the emergency department visits for asthma per 10,000 people for the three-year period from 2011 to 2013 (Figure 1). These data were extracted from the CalEnviroScreen 3.0, which was updated in 2018. The CalEnviroScreen 3.0 refers to an environmental health screening tool for the communities in California. It was designed not only to evaluate multiple pollutants and stressors at the local but also to assist local agencies in carrying out their environmental justice missions [16]. Based on hospitals’ emergency medical service report to California Office of Statewide Health Planning and Development (OSHPD), the CalEnviroScreen 3.0 provides the data by census tract. It is noteworthy that the dependent variable differs from the number of asthma patients. Due to the lack of individual asthma patient data, this paper employs the emergency department visit data. Considerable research employed asthma emergency room visit data as a proxy variable [8,17,18,19,20] Furthermore, some research reports that built environment presents a stronger correlation with severe asthma than with asthma morbidity [21,22]. Hypothesizing that asthma patients who visited emergency rooms experience severe asthma, the dependent variable can appropriately provide the insight of the relationship between asthma morbidity and greenspace.

Independent variables include three factors that represent not only the quantity of trees, but also the characteristics and configuration of tree patches (Table 1). They include TreeCov, TreeClus, and TreeAgr. The variable, TreeCov, refers to the areas covered by tree crowns and/or clustered canopies (TreeCov). This is the variable that represents the overall quantity of trees and that considerable research addressed [8,9]. This paper measured the median value of tree patch sizes in each census tract (TreeClus). It is reasonable to hypothesize that larger patches of trees (extended and connected canopy of trees) have a stronger capability to capture particles on their leaf surface, than smaller patches or an individual tree does. Since the values of tree size that are measured appear to be a skewed distribution rather than a normal distribution, median rather than mean was selected.

Another characteristic of tree path measured is the nearest neighbor index (NNI) of tree patches (TreeAgr). This paper adopts this variable under the assumption that the formations or configurations of tree patches can be an influential factor on asthma morbidity. The NNI indicates whether the distribution of tree patches is clustered or dispersed. It is a ratio of distance between each feature centroid and its nearest neighbor’s centroid location, which is a hypothetical random distribution with the same number of features covering the same total area [23]. If the NNI is less than 1, the pattern represents clustering, while an index greater than 1 indicates dispersion. In other words, the smaller NNI is, the more aggregated the pattern of tree patches is.

This paper also includes three open/greenspace variables that comprehensively capture green infrastructure in an urban environment. They include private, semi-private, and public greenspaces, which became three open/greenspace variables, PrvtGrn, SemiGrn, and GrnRec, respectively. Including front/back yards and gardens of residential areas, as well as landscaped areas in commercial, office, and industrial facilities, private greenspace (PrvtGrn) represents green and landscaped areas within privately owned properties. These spaces are relatively small scale and belong to private properties but are commonly found within close proximity to people’s everyday life. Semi-public greenspace (SemiGrn) indicates the greenspace of the facilities that have a certain level of restriction on the public access. They mainly include golf courses, schools, cemeteries, and agricultural lands. These facilities normally provide the public with a large scale of open/greenspaces in an urban environment, but people’s free access to the facilities is not warranted. Public greenspace (GrnRec) refers to greenspace which is largely accessible by the public, including local and regional parks and recreational areas. This greenspace offers not only green/open space but also opportunities for the public to engage in physical activities and exercise.

In order to compute these variables, this paper acquired the tree canopy and high-resolution land cover data from Los Angeles Regional Imagery Acquisition Consortium (LARIAC). LARIAC captured tree canopy using Light Detection and Ranging (LiDAR) technology, which allow creating high resolution digital surface models (DSM) and extracting the tree canopy from DSM. The tree canopy data were in the format of raster at 2 feet by 2 feet resolution. This paper captured open/greenspaces in the urban environment with the high-resolution land cover data (0.2286 m by 0.2286 m) from LARIAC and the parcel-based land use data from Southern California Association of Governments (SCAG).

**Figure 2:**
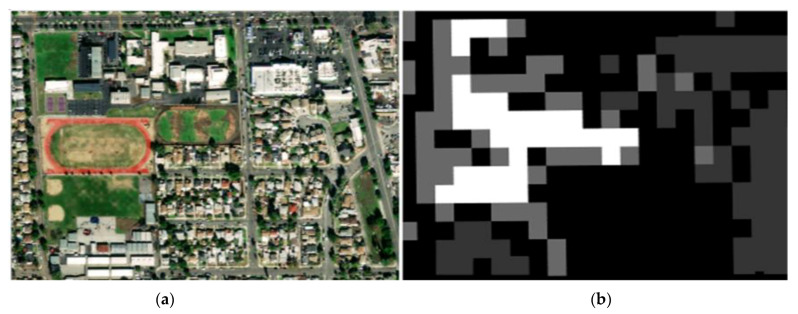

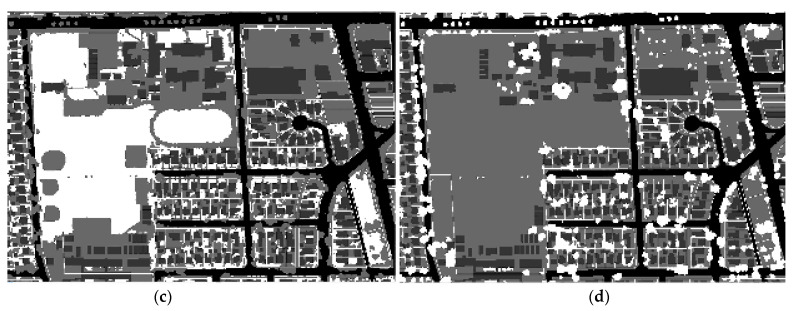
Example of tree and open/greenspace data. Note: (**a**) an aerial image that illustrates an actual urban area with diverse land uses with 0.3 m by 0.3 m resolution (source: ESRI), (**b**) conventional land cover data from Landsat satellite imagery that captures greenspaces (white) with 30 m by 30 m resolution (source: National Land Cover Database), (**c**) LARIAC land cover data that capture greenspaces (white), and (**d**) LARIAC tree data that capture trees (white) with 0.2286 m by 0.2286 m resolution (source: LARIAC).

By overlaying these two datasets, it was possible to precisely extract greenspaces within properties designated for a specific land use type. Extensive existing research detected greenspace with land cover data from satellite images [8,9,12,13]. The resolution of the land cover data is normally 30 m by 30 m or 10 m by 10 m. For example, the Normalized Difference Vegetation Index (NDVI) data derived from satellite imagery data, whose resolution is typically 30 m by 30 m. The LARIAC data allow delineating the shape and area of tree and greenspaces at a much finer level than the conventional satellite imagery data. For instance, the LARIAC land cover data are about 1914 and 17,222 times finer than 10 m by 10 m and 30 m by 30 m satellite imagery data, respectively (Figure 2).

Since the socio-demographic background of asthma patients is often mentioned as one of most significant risk factors of asthma in previous studies [24,25], several socio-demographic characteristics at the geographic level were defined as control variables. Given the direct association between air pollution and asthma morbidity, it is also fairly reasonable to control outdoor air quality variables. Thus, this model includes these variables. Lastly, all of socio-demographic factors were based on the 2016 American Community Survey U.S. (5-year estimates), while the air quality variables were extracted from CalEnviroScreen 3.0.

Using the variables, this paper constructs two spatial regression methods: a spatial lag (SL) and a spatial error (SE) model. Whereas the SL model presupposes that dependencies lie straightly in the levels of the dependent variable, the SE model treats primarily the spatial correlation as a problem that should be fixed. It is very similar to how statistical approaches often treat temporal serial correlation as something to be eliminated and solely regarded as an estimation problem [26]. The SL and SE models can be expressed using the following formulas, respectively.

*y* = *pWy* + *Xβ* + *ε* (1)

where

*y* = a vector of observations on the dependent variable;
*Wy* = a spatially lagged dependent variable for the weight matrix, W;
*X* = a matrix of observations on the explanatory variables;
*ε* = a vector of error terms;
*p*, *β* = parameters.

*y* = *Xβ* + *ε with ε* = *λWε* + *u* (2)

where

*y* = a vector of observations on the dependent variable;
*W* = the spatial weight matrix;
*X* = a matrix of observations on the explanatory variables;
*ε* = a vector of spatially autocorrelated error terms;
*u* = a vector of errors;
*λ*, *β* = parameters.

The primary reason for employing the spatial regression methods is spatial autocorrelation. In previous environmental health studies, less consideration of the spatial autocorrelation inherent to geo-referenced data has been often reported as a possible methodological limitation [27]. Due to the nature of the unit of analysis (i.e., census tract), the tendency of spatial autocorrelation and/or heterogeneity is often reported. In other words, a value observed in one location not only relies on the values observed at neighboring locations but also tends to be more clustered than distanced ones. Ignoring this spatial data issue in employing a statistical model might lead to biased results [27,28]. A Moran’s I test on the residuals from the OLS regression model for the correlation confirmed a significant spatial autocorrelation (0.782 of Moran’s Index, 66.734 of z-score, and 0.000 of *p*-value). Based on this result, we verified that, rather than OLS model, spatial regression model is a more reasonable and relevant method for this paper. This is because spatial regression models can explicitly incorporate the mechanisms underlying the geo-spatial dependency of observed data in the real world.

## 3. Analysis and Findings

Prior to spatial regression modeling, this paper summarized the descriptive statistics for the variables (Table 2). Possible collinearity of independent variables might affect the relationships between the explanatory variables and asthma morbidity. Given this concern about potential bias due to multicollinearity, we conducted VIF test and then confirmed that all VIF values are below 5. Based on this result, we reject the possibility of multicollinearity between explanatory variables [29] The majority of the tree and open/greenspace variable values present relatively small standard deviation values, except semi-public greenspaces (SemiGrn) and public open/greenspaces (GrnRec). Small standard deviation values mention that the variables are consistent in the study area with less variation, since it denotes that the variables observed are closely distributed around the mean value. The relatively large standard deviations for SemiGrn and GrnTec indicate the uneven spatial distribution of semi-public and public open/greenspaces. For example, semi-public greenspaces are concentrated around the periphery of and between major cities, due to urban land use patterns. Similarly, the ratio of public open/greenspaces in dense urban areas and along major transportation corridors is extremely low, while they are distributed in suburban areas.

Overall, the R-squared and log likelihood values from the two models indicate a good model fit. The R-squared value of the SE model, 0.785, presented a slightly better general model fit in comparison with the one of the SL model (0.718). Since the best model is the model with the lowest AIC score, the AIC scores are consistent with the R-squared values (Table 3). The outputs from both models are consistent. While the SL model confirms the correlation of three variables, PrvtGRN, SnrPop, and Ozone, with the dependent variable at a statistically significant level (0.10 level), the correlation at the level is not found from the SE model. Otherwise, the output of the two models remains the same.

The outputs of both models suggest interesting findings and discussion topics. The SL model displays the relationship between the dependent variable and four tree /greenspace variables: TreeCov, TreeClus, TreeAgr, and PrvtGrn. The significant socio-demographic variables from both models are the poverty rate (PovRt), the ratio of the African American population (EthGrp), and children population (ChldPop), which indicate positive correlations with asthma morbidity. The output confirms previous research that identified the correlation between asthma incidence and the patients’ socio-economic cohorts, including the children, low income, and minority population [24,30]. Consistent with the earlier studies, the result also indicates the positive correlations of asthma emergency room visits with poverty rate, the ratio of the African American population, and the ratio of children population [22,31]. As expected, the air quality variables also present a positive correlation with asthma emergency room visits. A positive correlation between PM 2.5 and the dependent variable at a statistically significant level is identified from both models, while the SL model only presents the correlation of ozone with the dependent variable.

By controlling the socio-demographic and air quality factors, the outputs from the models suggest clear correlations of tree and greenspace with asthma morbidity in Los Angeles County. The findings infer a strong relationship between trees and asthma morbidity. All of the three tree variables present a correlation with asthma emergency room visits at a statistically significant level. The models also indicate that the ratio of the areas covered by tree canopies (TreeCov) negatively correlates with asthma morbidity. Overall, this finding is consistent with the previous research at the ecological level, which suggested the positive influence of trees and greenspaces on asthma [8,9,10,32,33].

However, the models add a new perspective on the relationship between the characteristics of tree cluster and asthma morbidity. Both models present a negative correlation of TreeClus with the dependent variable. In other words, there are a smaller number of asthma emergency room visits in the areas with larger clusters of trees. This suggests that larger patches of trees (extended and connected canopy of trees) have a stronger capability to capture particles on their leaf surface than smaller patches or an individual tree does. This also suggests that mature trees with a relatively large canopy are more effective than smaller trees in terms of purifying the air. The improved air quality by this critical mass of tree patch probably contributes to the reduction of asthma emergency room visits.

In like manner, the positive correlation between TreeAgr and asthma morbidity illustrates that the aggregated tree patches have positive impacts on asthma. This implies that the configuration of trees, as well as the quantity of trees, is also relevant to asthma morbidity. The finding indicates that people have fewer emergency room visits for asthma in areas where the pattern of trees is aggregated rather than dispersed. In the consideration of the finding that asthma morbidity is associated with the size of the tree patch, it is clear that large, aggregated tree patches positively contribute to asthma morbidity.

Another interesting finding is the negative correlation between the variable, PrvtGrn, and the dependent variable confirmed by the SL model. In other words, the model indicates that people in the areas with more private greenspaces and landscaped areas have less chance of making have asthma emergency room visits. However, none of the models identifies the correlation of the other variables, GrnRec and SemiGrn, with asthma morbidity. Semi-public and public open/greenspaces do not contribute to asthma morbidity.

In comparison to semi-public and public open/greenspace (e.g., golf course, school’s playgrounds, parks, and cemeteries), individual private greenspaces (e.g., yards of residential properties and landscaped spaces in commercial properties) occupy a small geographical extent. Private greenspaces are instead available within close proximity of people’s everyday life and activities. This finding implies that the accumulation of small-scale spaces around daily activities is an important contributing factor to asthma. The large scale open/greenspaces, such as golf courses, parks, and school fields do not strongly associate with asthma morbidity, unless perhaps they are close enough to people.

## 4. Discussion

Findings from analytic models provide insights into the current environmental projects of many cities based on a better understanding of the complete relationship between greenspaces/trees in the neighborhood level and asthma morbidity and for exploring air-quality mitigation policies from the perspective of asthma prevalence. For example, the Million Tree Initiative started by the City of Los Angeles in 2006 is a project that has the goal of adding a million more trees to its existing forests. This project was adopted by many cities in the U.S., like Denver and New York City, as well as international cities as London and Shanghai [34]. The original purpose of the project was to mitigate climate change and to reduce air pollution, especially carbon dioxide, by expanding urban forestry. Therefore, when planning and assessing the project, the public health benefits of the project were unfortunately underestimated, while emphasizing its benefits in energy saving, air quality, and aesthetics [35]. As the models indicate, the increase of tree and green spaces can contribute to the improvement of asthma-related public health. Therefore, when planning a project like the Million Tree Initiative, it is important for municipalities to increase the public health benefits by strategically planting trees in the areas with the high concentration of asthma patients.

Speaking of the strategic locations of urban forestry, a considerable policy recommendation and guidance is to maintain 500-feet distance between the development of new schools, housing or other sensitive land uses and major highways [36]. Due the significant negative contribution of major highways to air quality, consequentially to asthma morbidity [31,37], governments require the buffer distance. Based on the results from our empirical models, in tandem with maintaining the buffer distance, planting trees in these buffer areas will help to improve poor air quality and hence to reduce asthma morbidity. Thus, in practice, targeting these areas for creating urban forestry is crucial for local governments and transportation agencies to mitigate air pollution.

When creating urban forestry, it would be ideal to strategically select the sites of urban forestry in a way that they are clustered and aggregated. This is because aggregated rather than dispersed trees contribute to the relief of asthma. Furthermore, it is important to plant trees in a clustered form. Although larger trees have a stronger positive impact on asthma, it is not easy to start by planting mature, large trees. Therefore, it is necessary to have a strategic, long-term vision that plants trees close enough, so that the canopy of the trees will be extended and connected in the future.

The findings indicate the positive contributions of private green spaces like front/back yards and landscaped areas to the reduction of asthma morbidity. The private green spaces are small, but proximate greenspaces in comparison to the semi- and public greenspaces. Therefore, it is important to create small-scale greenspaces near people’s everyday activities. For example, a pocket park can be an excellent alternative. A pocket park is a small park for the general public, which is typically created on a single vacant lot or on small, irregular pieces of land, especially in dense urban environments. Another example is the conversion of surface parking lots to greenspaces. With the movement of sustainable parking management that suggests the reduction of surface parking lots [38], cities have been putting significant efforts into turning parking lots into parks, community gardens, and/or other greenspaces. These efforts will have a positive effect on the reduction of asthma morbidity.

## 5. Conclusions

We offered an investigation of the role of neighborhood tree and greenspace, particularly in terms of the area, configuration, and feature, on asthma patients. Employing high-resolution land cover and tree data derived from advanced LiDAR technologies, this paper pays special attention to improving the measurement of tree and greenspace factors. The data facilitate the accurate measurement of not only the area of neighborhood tree and greenspaces but also the configuration and characteristics. The results from two spatial regression models indicated that the relationships between neighborhood tree/greenspaces and asthma are much more complicated than the well-known effect of the quantity of urban greenspace acreages on asthma incidence. In addition to the areas of greenspaces, which are conventionally identified as a contributing factor to asthma, the findings suggest that the configuration of trees and the characteristics of greenspaces at the neighborhood level influence asthma morbidity in Los Angeles County. They include the size of the tree canopy, the level of tree cluster, and private small-scale neighborhood greenspaces.

Overall, the relationship between greenspace and asthma morbidity is a topic that much research has conventionally addressed. However, the relationship can be articulated by new high-resolution spatial data, which become more accessible by the latest advances in computer technologies and geo-spatial science. We expect that future studies with the emergence of big data and relevant analytic technologies will help us to reach more scientifically generalized and convincing empirical evidence. In addition to improving the accuracy of measurement on land cover, this trend can make it possible to introduce new variables and perspectives to the field of greenspace and asthma morbidity research. As an exemplary case, this research demonstrates the contribution of advanced spatial data to asthma morbidity-related literature. Although this paper articulates the measurement of multiple tree and greenspace factors, there is a limitation that should be addressed by future studies. The limitation is the aggregated asthma patient data by census tract. It would be ideal to identify the location of individual asthma patients and analyze the ambient green environment by the individual location. However, this paper employed the aggregated asthma patient data, mainly due to the limitation of individual asthma patient data availability. Therefore, it would be ideal for future research to employ asthma patient data at the individual level.

## Figures and Tables

**Table 1:** List of variables.

Variables	Definitions	Descriptions	Sources
**Dependent Variable**			
Asthma	The emergency department visits for asthma per 10,000 people by census tract (patients’ residential location basis, 3-year averages between 2011 and 2013)		CalEnviroScreen 3.0
**Tree Variables**			
TreeCov	Areas covered by trees	The percent of census tract areas covered by tree canopy (%)	Los Angeles Regional Imagery Acquisition Consortium
TreeClus	The size of tree patch	The median size of the clustered tree patch (square feet)	
TreeAgr	Nearest neighbor index (NNI)	The level of cluster (or dispersion) of tree patches	
**Green/Open Space Variables**			
PrvtGrn	Private urban greenspace	The percent of census tract areas occupied by garden/landscape space of urban land use types including residential, commercial, office, and industrial (%)	Los Angeles Regional Imagery Acquisition Consortium (LARIAC)
GrnRec	Greenspace in recreational areas	The percent of census tract areas occupied by greenspace, which is largely accessible by the public, including parks and recreational areas (%)	
SemiGrn	Semi-public greenspace	The percent of census tract areas occupied by greenspace, which is not accessible by the public, including golf courses, educational facilities, and cemeteries, as well as agricultural lands (%)	
**Socio-Demographic Variables**			
PovRt	Poverty rate	The percent of households whose income in the past 12 months was below the poverty level (%)	The American Community Survey (2016; 5-year estimates)
EduAtn	Education attainment	The percent of population with high school degree or lower (%)	
EtnGrp	Ethnical background	The percent of the African American population (%)	
ChldPop	Children population	The percent of children population whose age is 10 or under (%)	
SnrPop	Elderly population	The percent of the elderly population whose age is 65 or above (%)	
**Air Quality Variables**			
PM2.5	Particulate matter 2.5	The average of particulate matter 2.5 concentration from the years 2011 to 2013	CalEnviroScreen 3.0
Ozone	Level of ozone	The average daily maximum ozone concentration for the years 2011 to 2013	

**Table 2:** Descriptive statistics of variables.

Variables	Minimum	Maximum	Mean	Std. Deviation	VIF
Asthma	0.00	154.14	52.07	25.36	-
Tree Variables					
TreeCov	0.00	0.42	0.11	0.06	4.666
TreeClus	8.00	212.00	76.38	21.33	4.413
TreeAgr	0.20	1.14	0.81	0.15	1.088
Open/Greenspace Variables					
PrvtGrn	0.00	0.31	0.09	0.05	1.567
GrnRec	0.00	0.53	0.01	0.02	1.057
SemiGrn	0.00	0.85	0.01	0.04	1.046
Socio-Demographic Variables					
PovRt	0.00	100.00	18.82	13.01	1.395
EduAtn	0.00	100.00	1.79	21.90	1.013
EtnGrp	0.00	89.98	8.17	13.08	1.162
ChldPop	0.00	40.60	12.80	4.26	1.626
SnrPop	0.00	100.00	11.02	6.05	1.537
Air Quality Variables					
PM2.5	5.34	12.89	11.53	1.28	1.726
Ozone	0.04	0.07	0.05	0.01	1.720

Note: Std. Deviation = standard deviation.

**Table 3:** The outputs of spatial regression models.

Variables	SL Model				SE Model				OLS Model	
	Coef.	Standardized	S. E.	Z	Coef.	Standardized	S. E.	Z	Coef.	S. E.
Constant	7.586	24.613	6.376	1.190	48.153	50.304	15.520	3.103	52.075	0.375
Tree Variables										
TreeCov	−44.692	−3.660	5.702	*** −7.838	−52.911	−2.054	7.196	*** −7.353	−1.616	0.809
TreeClus	−0.034	−1.830	0.016	** −2.202	−0.033	−0.820	0.016	** −1.979	−3.660	0.787
TreeAgr	15.687	1.616	2.693	*** 5.824	15.232	2.138	2.908	*** 5.238	0.309	0.397
Open/Greenspace Variables										
PrvtGrn	−12.998	−1.232	6.954	* −1.869	13.428	0.357	8.178	1.642	−0.166	0.469
GrnRec	3.876	0.169	12.406	0.312	9.543	0.231	10.126	0.942	0.265	0.385
SemiGrn	−1.567	0.033	7.267	−0.216	1.014	0.031	5.971	0.170	0.183	0.383
Socio-demographic Variables										
PovRt	0.172	2.059	0.027	*** 6.406	0.061	0.746	0.024	** 2.531	4.485	0.443
EduAtn	−0.003	−0.037	0.013	−0.231	−0.008	−0.208	0.009	−0.955	0.546	0.377
EtnGrp	0.365	5.018	0.025	*** 14.891	0.280	3.733	0.029	*** 9.647	9.763	0.404
ChldPop	0.843	3.612	0.087	*** 9.680	1.045	4.503	0.096	*** 10.906	7.490	0.478
SnrPop	−0.105	−0.623	0.058	* −1.798	−0.073	−0.524	0.046	−1.585	−0.226	0.464
Air Quality Variables										
PM2.5	0.583	−0.764	0.320	* 1.822	1.711	−2.105	0.796	** 2.149	−2.138	0.492
Ozone	114.113	0.528	65.976	* 1.730	21.651	−0.200	166.955	0.130	0.215	0.491
R-Squared	0.718				0.785				0.501	
Log likelihood	−9341.340				−9184.231				N/A	
Akaike info Criterion (AIC)	18714.7				18398.5				N/A	
Lag coefficient	(Rho) 0.541				(Lambda) 0.797				N/A	

Note: *, **, *** Correlations are significant at the 0.10, 0.05, and 0.01 levels, respectively (2-tailed) Coef. = Coefficient; Standardized = Standardized coefficient; S. E. = Standard error; Z = Z-value.

## Data Availability

Party Data. Restrictions apply to the availability of these data. The tree-canopy and high-resolution land cover data were obtained from Los Angeles Regional Imagery Acquisition Consortium (LARIAC) and are available from https://lariac-lacounty.hub.arcgis.com/ (accessed on 30 September 2019) with the permission of LARIAC. The land use data were obtained from Southern California Association of Governments (SCAG) and are available from https://scag.ca.gov/ (accessed on 30 September 2019) with the permission of SCAG. The other data used in the paper are publicly available. This includes CalEnviroScreen 3.0. (available from https://oehha.ca.gov/calenviroscreen/report/calenviroscreen−30) (accessed on 30 September 2019) and U.S. Census data (available from https://www.census.gov/) (accessed on 30 September 2019).
